# Single‐nucleus transcriptomic and chromatin accessibility analysis of rat hippocampal cells following amyloid‐beta oligomers injections

**DOI:** 10.1002/alz70855_103834

**Published:** 2025-12-24

**Authors:** Tra‐My Vu, Klarissa Leduc, Martin Newton, Jonathan Brouillette

**Affiliations:** ^1^ Université de Montréal, Montréal, QC, Canada

## Abstract

**Background:**

Alzheimer's disease (AD) is a neurodegenerative disease characterized primarily by the aggregation of amyloid‐beta (Aß) oligomers and neurofibrillary tangles. Accumulation of Aß oligomers is detected 10 to 15 years before the appearance of the first clinical symptoms of AD. Studies have shown that Aß oligomers are involved in synaptic and neuronal losses observed in the hippocampus, a brain region critically involved in memory that is affected in the early stages of AD. Thus, we are interested to study the impact of Aß oligomers on the different cell types present in the hippocampus using a rat model via single‐nucleus transcriptomic and chromatin accessibility profiling.

**Method:**

Aß oligomers were injected daily into the hippocampus for 0 (control), 2, 4 or 6 days. Subsequently, the brains were collected and flash freeze. Cells located directly under the injection site were cryosection at 50 micrometers then collected by laser microdissection. The nucleus was isolated, and we performed a transcriptomic and chromatin accessibility analysis simultaneously on each of these nucleus of neurons, astrocytes, oligodendrocytes, pericytes, endothelial cells and microglia, using the single nucleus RNA‐sequencing and single nucleus ATAC‐sequencing technics (10xGenomics).

**Result:**

Neuronal loss was observed at the granular layer of the dentate gyrus caused by Aß oligomers injection. Indeed, we observed a significant accumulation of Aß in rat's hippocampus which were consecutively injected with Aß 6 consecutive days, compared to 2 and 4 consecutive days of injection. The immunofluorescence's results with NeuN and GFAP showed a neuronal loss after repetitive injections. Moreover, our test sample's sequencing profiles identified different clusters with different interesting gene expression changes over time.

**Conclusion:**

This study will allow us to determine the gene expression and epigenetic changes induced specifically by Aß oligomers in each hippocampal cell type during the progression of Aß pathology. This could pave the way to find new therapeutic targets efficiently in the early stages of AD.

